# Benchmarking Food and Beverage Companies on Obesity Prevention and Nutrition Policies: Evaluation of the BIA-Obesity Australia Initiative, 2017-2019

**DOI:** 10.34172/ijhpm.2020.147

**Published:** 2020-08-22

**Authors:** Ella Robinson, Miranda R. Blake, Gary Sacks

**Affiliations:** Global Obesity Centre (GLOBE), Institute for Health Transformation, Deakin University, Geelong, VIC, Australia.

**Keywords:** Benchmarking Food Companies, Obesity Prevention, Nutrition Policy, Organisational Change, Australia

## Abstract

**Background:** The potential role of the food and beverage industry in addressing diet-related disease is much debated, particularly amidst evidence of the targeted strategies, including voluntary self-regulation, used by the industry to influence policy in their favour. At the same time, the need for more comprehensive action to address unhealthy diets has led to a focus on increasing the accountability of different stakeholders. However, there has been limited evaluation of the impact of accountability initiatives on food and beverage company policies and practices. This study evaluated the impact of the BIA-Obesity (Business Impact Assessment – Obesity and population nutrition) Australia Initiative that benchmarked major Australian food and beverage companies on their nutrition-related policies.

**Methods:** Evaluation was conducted against the pre-specified logic model for BIA-Obesity and established frameworks for analysing organisational change and corporate political activity. Outcomes evaluated included company engagement with the Initiative, level of media coverage, and impact of the Initiative on company policies and practices based on the perspectives of company representatives. A mixed methods design was employed, including surveys and in-depth interviews with company representatives, and media reports.

**Results:** Approximately half of invited companies participated in the evaluation of the BIA-Obesity Australia Initiative. A number of company representatives indicated that the Initiative had influenced their company’s nutrition policies, strategies, and disclosure practices, and had raised their company’s awareness of the importance of addressing nutrition issues.

**Conclusion:** Company representatives perceive benchmarking and accountability initiatives as helpful for provoking improvements in nutrition-related policies and practices in their companies. However, the benefits of these initiatives need to be assessed in the context of the broader political and economic environment. Whilst the focus of accountability initiatives, such as BIA-Obesity, are on industry self-regulation efforts, they can also play an important role in drawing attention to the need for increased government regulation.

## Background

There is wide-spread recognition that a comprehensive societal approach is needed to address unhealthy diets and obesity.^1,2^ This comprehensive approach needs to include actions from government, industry and other stakeholders to create healthier food environments.^3^ Nevertheless, there have been few governments, including those in Australia, that have taken meaningful action to address unhealthy food environments, and progress has been slow to date.^2,4,5^ With respect to the food industry, the World Health Organization (WHO) and the United Nations have identified specific roles and actions that food and beverage companies can take to contribute to improving the healthiness of food environments.^6,7^ These include reducing the exposure of children to marketing of unhealthy foods, product reformulation and improved nutrition labelling.^7^ However, the potential role of the food industry in efforts to address diet-related disease is much debated,^8,9^ particularly amidst evidence that large food and beverage companies use a wide-range of strategies to influence policy and public opinion in their favour.^10,11^ Among these strategies, ‘policy substitution’ (whereby food and beverage companies develop voluntary policies and codes as an alternative to regulatory action) and ‘constituency building’ (in which companies strategically develop relationships with influential stakeholders) have been identified as key tactics used by companies in efforts to prevent or delay implementation of regulations that may negatively affect their profitability.^10,12,13^

Several authoritative reports related to obesity prevention have identified the importance of monitoring food industry commitments related to nutrition as part of accountability mechanisms.^2,14^ Globally, several initiatives monitor and benchmark the food and beverage industry in relation to nutrition and obesity. The non-profit Access to Nutrition Initiative (ATNI), which benchmarks the obesity and undernutrition related commitments, practices and performance of food and beverage manufacturers, is the most prominent global initiative in this area.^15^ The ATNI has had significant engagement from the food industry and the investment community, and has launched three global indexes (2013, 2016, 2018), as well as spotlight indexes in India and the United States.^16-18^ The International Network for Food and Obesity/NCDs Research, Monitoring and Action Support, a global network of public health researchers that aims to monitor and benchmark food environments globally,^19^ developed the BIA-Obesity (Business Impact Assessment – Obesity and population nutrition), based on the ATNI. The BIA-Obesity is a tool and process that aims to benchmark the food and beverage industry on their policies and commitments related to obesity prevention and population nutrition at the national level, with tailored indicators for food and beverage manufacturers, supermarkets, and quick service restaurants.^20^

The BIA-Obesity assesses companies (out of 100) across a range of indicators (54 indicators for food and beverage manufacturers, 73 for supermarkets, 56 for quick service restaurants) from 6 key policy domains, including (1) Corporate nutrition strategy; (2) Product formulation; (3) Nutrition labelling; (4) Promotion practices; (5) Product accessibility; and (6) Relationships with external groups.^21^ A company’s policies and commitments under each policy domain are scored based on their transparency, comprehensiveness and specificity. Companies are then provided with tailored recommendations on areas for improvement. In Australia, BIA-Obesity was first implemented over the period 2017-2018 (the BIA-Obesity Australia Initiative). As at June 2020, BIA-Obesity had also been implemented in several other countries, including New Zealand,^22^ Canada^23^ and Malaysia.^24^

Despite increasing focus on industry accountability initiatives with respect to health issues, and nutrition in particular,^15,25^ there has been no formal publicly available evaluation of the impact of these initiatives on food and beverage industry policies and practices. This study aimed to evaluate the impact of the BIA‐Obesity Australia Initiative on relevant company policies and practices.

## Key Messages

**Implications for policy makers**

- Company representatives report that benchmarking and accountability initiatives can be helpful for driving improvements in nutrition-related policies and practices.
- Large food and beverage companies differ in their commitment to health and nutrition issues, their competitive positioning with respect to health, and their stated willingness to change. Food and beverage companies should work towards strengthening their nutrition-related policies and practices, in line with public health recommendations.
- Where industry nutrition policies and practices are shown to be weak, benchmarking and accountability initiatives can serve to highlight the need for greater government regulation in the area of nutrition and obesity prevention.
- Policy-makers can play a role in strengthening industry self-regulatory commitments, through implementing reporting frameworks; time-bound, measurable targets; and sanctions for non-compliance.

**Implications for public**

The food and beverage industry can play a role in efforts to improve population diets and address obesity and non-communicable diseases. Benchmarking and accountability initiatives are one mechanism to stimulate change and encourage meaningful action from food and beverage companies towards improving population health. The public can express their support for companies to improve their nutrition-related policies and practices in public forums and through direct feedback to company executives. Moreover, the public has the power to influence food and beverage companies through their purchasing decisions and investment decision-making. The public can also join collective efforts to support the role of governments in addressing unhealthy diets and obesity.

## Methods

###  Overview

 This evaluation employed a convergent mixed methods approach to evaluate the impact of the BIA-Obesity Australia Initiative on relevant company policies and practices over the period February 2017-July 2019. Outcome measures for the evaluation were investigated using predominantly qualitative information from multiple sources. The primary source of data was in-depth interviews on the perspectives of company representatives from each sector of the food and beverage industry (food and beverage manufacturers, supermarkets and quick service restaurants). This was supported by surveys with company representatives and media reports. The methodology behind the use of each data source is described in greater detail below. See Figure for a timeline of the BIA-Obesity Australia Initiative and the related evaluation activities.

**Figure:**
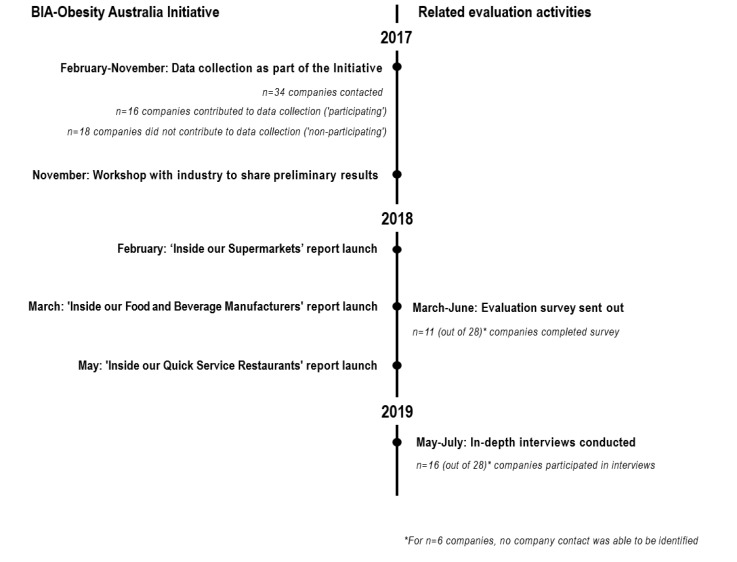
Timeline for the BIA-Obesity Australia Initiative and Related Evaluation Activities (2017-2019). Abbreviation: BIA-Obesity, Business Impact Assessment – Obesity and population nutrition.

A previously published logic model for BIA-Obesity,^21^ informed the design of the evaluation (see Supplementary file 1, Table S1). Key short- and medium-term outcome measures for the evaluation were identified through the logic model. These outcomes were evaluated at 2 years after initial engagement with food and beverage manufacturers, supermarkets and quick service restaurants, and 12-16 months after the launch of the key reports detailing the results of the Initiative. Outcome measures for the evaluation included: food and beverage company participation and engagement with the Initiative; media attention generated by the Initiative; impact of the Initiative on food and beverage company nutrition policies, commitments and disclosure practices related to obesity prevention and population nutrition. These outcome measures were chosen as they related directly to the impact of the BIA-Obesity Australia Initiative on food and beverage companies. The evaluation also sought company perspectives on the role of the public health community in supporting the food and beverage industry to address nutrition, perceptions of benchmarking effectiveness, and ways in which BIA-Obesity could be improved.

###  Theoretical Approach 

A pragmatic approach guided the survey and interview guide design,^26^ structured around the outcomes of interest. Concepts from organisational change theory, specifically the Content, Context, Process (CCP) framework,^27,28^ were used to guide the development of data collection tools. The CCP framework emphasises the importance of economic, social, political and cultural factors in driving organisational change and focuses on the interconnectedness of three components of the change process: content (the ‘what’), context (the ‘why’) and process (the ‘how’). The CCP framework was also used in analysing and interpreting the data, and is used to structure the ‘Discussion’ section of the manuscript.

###  Data Sources

####  Characteristics of Companies

Characteristics of companies involved in the BIA-Obesity Australia Initiative were assessed by company sector (food and beverage manufacturer, supermarket, and quick service restaurant), country of headquarters, overall score (out of 100) as part of the BIA-Obesity Australia Initiative, and the nature and healthiness of their product portfolio. Companies were specified as being headquartered: (*i*) Australia; (*ii*) New Zealand; (*iii*) Australia and New Zealand; or (*iv*) Internationally. Recently published data on the healthiness of major food and beverage manufacturers operating in Australia^29^ was used to compare the nutrient profile of products of companies involved in the Initiative, categorised using the Australian Government-endorsed Health Star Rating (HSR) front of pack labelling system. As part of that analysis, a company’s product portfolio was considered ‘healthy’ if it had a mean HSR of >3.5. The product portfolio assessment was not possible for quick service restaurants, as there was no comparable available dataset on average nutrient profile. Companies were categorised as ‘participating’ if they agreed to participate in the BIA-Obesity Australia Initiative by providing and validating information on company policies and commitments. All other companies were categorised as ‘non-participating,’ in which case their assessment as part of the BIA-Obesity Australia Initiative was based on publicly-available policy information only.

####  Extent of Media Coverage 

Data from Isentia, a well-established media monitoring and analysis company,^30^ was used to describe the media coverage related to the BIA-Obesity Australia Initiative. This included relevant print, television, radio, and online media coverage in the 2 weeks following the release of each of the three reports (‘Inside our Supermarkets’ report, ‘Inside our Food and Beverage Manufacturers’ report, and ‘Inside our Quick Service Restaurants’ report). These were identified using search terms that included the name of the reports, and the names of the university and lead researchers involved in the Initiative. This data was supplemented by an internet search, drawing on quotes from media articles and statements. Data were analysed using total cumulative audience reach and number of media items for each report launch.

####  Surveys With Company Representatives

 Invitations to complete an evaluation survey, administered online through SurveyMonkey, were sent via email to food and beverage companies that were involved in the Initiative (both participating and non-participating), where an appropriate company contact had been identified during the BIA-Obesity Australia Initiative. For 6 companies, an appropriate contact had not been identified during the BIA-Obesity Australia Initiative, and, as such, these companies were unable to be contacted (total number of companies contacted = 28). Evaluation surveys were sent out approximately one month after the launch of the reports detailing the results of the Initiative (early 2018). Representatives from 11 out of the 28 contacted companies participated in the evaluation survey. Evaluation survey responses were anonymous, however respondents were asked to specify the sector (supermarket, food and beverage manufacturer, quick service restaurant) in which the company operated. The ten item survey consisted of a mix of open ended and multiple choice survey questions focused on: (1) the influence of the Initiative on work practices, policies and resourcing; (2) participation in the Initiative; (3) willingness to participate in follow up assessments; and (4) company areas of strength and areas for improvement in relation to nutrition (see Table S2 for the evaluation survey). Multiple choice survey responses were analysed descriptively. Short written responses were transcribed and analysed deductively against outcome measures.

###  In-depth Interviews With Company Representatives

Semi-structured interviews were conducted over the phone between May-July 2019 (12-16 months following the launch of the BIA-Obesity Australia reports). Interview questions focused on: (1) company engagement with the Initiative; (2) changes to company policies, practices, and resourcing in response to the Initiative; (3) interviewee perceptions of benchmarking exercises, and the role of the public health community in driving food sector action; and (4) areas for improvement in relation to the assessment tool and process (see Table S3 for the interview guide). Food and beverage companies involved in the Initiative (both participating and non-participating) were invited to participate in an interview. Representatives from 16 out of the 28 invited companies for which we had contact information participated in the interviews. The majority of company representatives (‘representatives’) contacted had been the lead contributor or liaison for the Initiative. Interviews were audio recorded and transcribed verbatim. One company requested that they provide a written response. Transcribed interviews were analysed deductively, using a coding framework derived from *a priori* logic model outcomes, as well as openness to emerging themes. Data analysis and interpretation was conducted using NVivo 12 software.

###  Synthesis of Results

 Data from the different data sources were synthesised and reported together to evaluate relevant short- and medium-term outcomes (from the logic model). The key findings are presented in Results section in 4 parts. Section 1 presents the characteristics of companies involved in the BIA-Obesity Australia Initiative. Section 2 focuses on outcomes related to engagement with the Initiative, including media coverage and company perspectives on their experience of engagement. Section 3 focuses on outcomes related to the impact of the BIA-Obesity Australia Initiative on food and beverage companies. Section 4 analyses the perspectives offered by company representatives regarding engagement with the public health community.

## Results

###  1. Characteristics of Companies

 Thirty-four companies from 3 sectors of the food industry (food and non-alcoholic beverage manufacturers (n = 19), supermarkets (n = 4) and quick service restaurants (n = 11)) were included in the BIA-Obesity Australia Initiative (see Table for details on company characteristics). Sixteen companies were classified as ‘participating’ companies, as they contributed to data collection processes. The majority of companies included in the Initiative (participating and non-participating) were headquartered internationally (59%), followed by Australia (35%), Australia and New Zealand (3%), and New Zealand (3%). The proportion of companies headquartered internationally compared to in Australia was similar across participating and non-participating groups. The overall assessment score for companies varied widely. Scores ranged from 3/100 to 71/100 for food and beverage manufacturers, from 8/100 to 46/100 for supermarkets, and from 3/100 to 48/100 for quick service restaurants. Non-participating companies had a substantially lower median score overall (13/100), compared to participating companies (53/100). Two participating food and beverage manufacturers had product portfolios with a mean HSR above 3.5 stars (‘healthy’). All other manufacturers and supermarkets had a product portfolio with a mean HSR of less than 3.5 stars. All non-participating companies had a mean HSR of less than 3.5 stars. Companies typically had a diverse product portfolio, consisting of products from multiple categories, and there was no discernible pattern in participation across companies based on the categories dominating their product portfolios.

**Table:** Characteristics of “Participating” and “Non-participating” Companies Included as Part of the BIA-Obesity Australia Initiative (2017-2019)

**Participation in BIA-Obesity Australia Initiative**	**Company**	**Country of Headquarters**	**Top 3 Product Categories (by Number of Products in Portfolio)** ^a^	**BIA-Obesity Australia Assessment Score** **(Out of 100)** ^b^	**Mean HSR** **(SD)** ^a^
**Food and Beverage Manufacturers (n = 19)**					
Participating companies (n = 11)	Campbell Arnott's	United States	Bread and bakery products; Convenience foods; Non-alcoholic beverages	55	2.4 (1.4)
	Coca-Cola	United States	Non-alcoholic beverages	64	1.9 (1.1)
	Fonterra	New Zealand	Dairy; Edible oils and oil emulsions; Snack foods	51	2.0 (1.3)
	George Weston Foods	Australia	Bread and bakery products; Meat and meat products	44	2.8 (1.3)
	Lion Dairy and Drinks	Australia	Dairy; Non-alcoholic beverages	71	3.2 (1.3)
	Mars	United States	Sauces, dressings, spreads and dips; Confectionery; Cereal and grain products	64	2.3 (1.3)
	Nestlé	Switzerland	Cereal and grain products; Confectionery; Non-alcoholic beverages	69	2.6 (1.5)
	PepsiCo^c^	United States	Snack foods; Sauces, dressings, spreads and dips; Bread and bakery products	50	2.8 (0.9)
	Sanitarium	Australia and New Zealand	Cereal and grain products; Dairy; Special foods	64	4.1 (0.7)
	Simplot	United States	Fish and fish products; fruit and vegetables; Sauces, dressings, spreads and dips	62	3.8 (0.8)
	Unilever	The Netherlands/United Kingdom	Dairy; Convenience foods; Sauces, dressings, spreads and dips	68	2.7 (1.0)
Non-participating (n = 8)	Goodman Fielder	Australia	Bread and bakery products; Sauces, dressings, spreads and dips; Cereal and grain products	4	2.8 (1.1)
	Kellogg’s	United States	Cereal and grain products; Special foods	48	3.0 (1.1)
	Kraft Heinz	United States	Fruit and vegetables; Convenience foods; Non-alcoholic beverages	29	3.1 (1.2)
	McCain Foods	Canada	Convenience foods; Fruit and vegetables; Bread and bakery products	14	3.1 (1.1)
	Mondelēz	United States	Confectionery; Bread and bakery products; Dairy	42	1.2 (0.9)
	Parmalat	Italy	Dairy	3	3.1 (1.0)
	Schweppes	United States	Non-alcoholic beverages	8	1.7 (0.6)
	Tru Blu Beverages	Australia	Non-alcoholic beverages	9	2.4 (1.4)
**Supermarkets (n = 4)**					
Participating (n = 3)	Coles	Australia	Bread and bakery products; Fruit and vegetables; Meat and meat products^d^	40	3.0 (1.4)
	IGA	Australia	Dairy; Confectionery; Bread and bakery products^d^	8	2.6 (1.5)
	Woolworths	Australia	Fruit and vegetables; Bread and bakery products; Convenience foods^d^	46	3.2 (1.3)
Non-participating (n = 1)	ALDI	Germany	Dairy; Bread and bakery products; Fruit and vegetables	11	2.7 (1.3)
**Quick Service Restaurants (n = 11)**					
Participating (n = 2)	Nando’s	South Africa	Not applicable	31	Not available
	Subway	United States		48	
Non-participating (n = 9)	Chicken Treat	Australia		14	
	Domino’s	United States		3	
	Grill’d	Australia		10	
	Hungry Jacks	Australia		28	
	KFC	United States		41	
	McDonald’s	United States		42	
	Oporto	Australia		11	
	Pizza Hut	United States		27	
	Red Rooster	Australia		12	

Abbreviations: BIA-Obesity, Business Impact Assessment – Obesity and population nutrition; HSR, health star rating; SD, standard deviation. ^a^Based on ‘*FoodSwitch: State of the Food Supply’* 2019 report.^29^ Higher Health Star Rating indicates healthier products (0.5 = least healthy, 5 = most healthy). ^b^Based on BIA-Obesity Australia 2018 reports.^31-33^ ^c^ Refers to the Smith’s Snackfood division of PepsiCo only. Product category and HSR data for the beverage division of PepsiCo was not included in the ‘ *FoodSwitch: State of the Food Supply’* 2019 report. ^d^Refers to supermarket ‘own brand’ products only.

 Representatives from 11 companies (out of 28 contacted) participated in the evaluation survey that directly followed the launch of the key reports, and representatives from 16 companies (out of 28 contacted) participated in in-depth interviews. There were low levels of participation in the evaluation survey (n = 2 out of 11) from the quick service restaurant sector and low levels of participation in the in-depth interviews (n = 3 out of 16) from companies that did not participate in the initial data collection for the BIA-Obesity Australia Initiative. Roles for interview participants were varied. The majority of respondents were from nutrition, health or wellbeing roles (n = 7), followed by external/public affairs or relations (n = 3), research and development (n = 3), regulatory or policy (n = 2), corporate responsibility or environment (n = 2), and marketing (n = 1).

###  2. Engagement With the BIA-Obesity Australia Initiative

####  2.1. Media Coverage and Public Response From Companies

The launch of the reports received widespread media coverage at a national level in Australia. Overall, the reports generated 333 media items across print, online and television media. The ‘Inside our Supermarkets’ report received the highest number of media items (140), with an estimated audience of ~9.7 million people, followed by the ‘Inside our Quick Service Restaurants’ report with 127 media items and an estimated audience of ~7.0 million people, and the ‘Inside our Food and Beverage Manufacturers’ report with 66 items and an estimated audience of ~5.0 million people.^34^ The highest volume of coverage for the ‘Inside our Supermarkets’ report was television (TV), whilst for the ‘Inside our Quick Service Restaurants’ report, the highest volume of coverage was for online news. ‘Inside our Food and Beverage Manufacturers’ received a high volume of coverage across TV, print and online channels. For each launch, media reporting was generally focused on the bottom performers within each sector and the need for increased government regulation.

 At least 11 companies responded publicly to the launch of the reports. This included at least 6 positive responses, in which companies reaffirmed their commitment to nutrition and health, and at least 5 negative responses, including an instance of a company publicly threatening legal action against the report authors. For more details on the public response from companies, refer to Appendix S1.

####  2.2. Company Engagement With the Initiative

 In the interviews, representatives reported that various levels of their business had been aware of the BIA-Obesity Australia Initiative. For participating companies, many representatives noted that senior business leaders (eg, executive level, division directors) had been engaged, supportive or involved in the Initiative to some degree. Several representatives reported that they engaged senior managers within their business, and noted that they had shared the findings of the report at senior forums, leadership team meetings, and various other channels throughout the business. Some representatives commented on the extensive media surrounding the reports, observing that the media coverage was well ‘noticed’ by the company.

*“I think what was definitely recognised is the ranking and the benchmarking and how we fell out compared to our competitors”* – Company representative (research and development role).

 Representatives reported several reasons for their participation in the research. Across participating companies, stated reasons included the company’s existing commitment or focus on nutrition and health, a desire to be transparent with their policies, company values around improving nutrition and health, and a response to the expectations and needs of customers.

*“Through our corporate social responsibility and just the values of our business, we’re aligned to the principles [of this research] and [want to] uncover and improve that baseline in obesity” – *Company representative (corporate responsibility/environment role).

 Representatives from non-participating companies stated their reasons for not participating as being due to the company’s limited engagement with external stakeholders at the time (n = 1), a lack of available resources to participate (n = 1), and being overburdened with various survey requests (n = 1).

 From the surveys, most representatives (n = 7/11) agreed that it would be important to repeat the Initiative in order to monitor progress over time, and that participation in the Initiative was a good use of their time (see Figure S1). The majority of representatives (n = 8/11) thought that two or more years was an appropriate timeframe to repeat the Initiative and almost all representatives (n = 9/10) said that they would be willing to participate in future initiatives (see Table S4). A small number of interviewees raised concerns around the large amount of time needed to assist with data collection in relation to the Initiative.

###  3. Impact of the BIA-Obesity Australia Initiative on Food and Beverage Companies 

####  3.1. Changes to Company Approach to Addressing Nutrition and Health

 In the surveys, several representatives reported that the Initiative had led to changes within their company, including: implementing a formal process for tracking progress in relation to nutrition, reinforcing that health was a priority for the company, and prioritising reformulation after realising the progress that competitors had made.

*“Reformulation has certainly taken priority when learning other competitors are well ahead on this” – *Company representative (nutrition/health/wellbeing role).

 In the interviews, a number of representatives from participating companies reported that the Initiative had helped to raise the priority of nutrition and health within the business, had helped to put nutrition and health onto the agenda, including the executive agenda, or had strengthened the company’s focus on nutrition and health. Several representatives said that the BIA-Obesity Australia reports had helped to reinforce areas in which the company was either doing well or needed to focus its future efforts. More specifically, representatives from participating companies reported that the Initiative had helped to: formally document the company’s nutrition strategy; advocate internally around the need for a formal nutrition policy; inform the company’s nutrition strategy; provoke a review of the healthiness of some products and associated nutrient-profiling criteria; and, drive new product reformulation goals.

*“We’ve done a review of our nutrition criteria. A lot of that, I would say, is really being generated around that [BIA-Obesity] feedback from an independent perspective that could be presented to the [executive] that then drove some of that” – *Company representative (nutrition/health/wellbeing role).

 In the interviews, several representatives, including two from non-participating companies, reported that the Initiative had limited impact on their approach to nutrition and health. This was predominantly due to the company already having a significant body of work or focus on nutrition and health. Two other representatives from internationally headquartered companies also noted that policies were set at the global level, and as such there was little room for their company to make changes locally.

####  3.2. Changes to Disclosure and Transparency Practices

 In the interviews, a number of representatives from participating companies noted the Initiative had highlighted the value in being more transparent and/or better communicating policies, commitments or actions to external stakeholders and their consumers. Several representatives stated that as a result of the Initiative they had made changes to their website, communicated externally on past achievements, or had discussions around the need for transparency, including articulating internal policies and strategies on their corporate website.

*“I think the report definitely highlighted, because you were looking at what are our external facing commitments and policies as your first step, it did highlight that having those external facing, or even if they’re not on a website, actually sharing what they [nutrition policies] are with government and with external stakeholders is really important” – *Company representative (nutrition/health/wellbeing role).

 Of note, all representatives from non-participating companies reported that the Initiative had helped the company to be more transparent, including through making the company re-evaluate their global communication strategy; helping the company to be more proactive in communicating their activities; and being a catalyst for communicating some of their internal commitments on their website:

“ *So, since the [BIA-Obesity Australia] report, within a few months of it, the first thing we did was update our corporate website to include a nutrition section” – *Company representative (regulatory/policy role).

 Representatives from participating and non-participating companies identified barriers associated with transparency, including accountability associated with public-facing targets if they were not achieved; limited resourcing for external communication; and concerns regarding sharing internal policies and commitments with competitors and consumers.

 Several representatives from participating companies reported that the Initiative had limited or no impact on their disclosure or transparency practices. Most commonly, this was due to companies stating that they were already being transparent with their policies and commitments.

####  3.3. Changes to Resourcing for Nutrition and Health

 In the surveys, two representatives reported that the Initiative had helped to leverage more resources for nutrition and health, whereas others indicated this was not the case (see Figure S1). This was consistent with the interviews in which a small number of representatives noted that the Initiative had contributed to more resources (new, dedicated internal roles) for nutrition and health. Nevertheless, most representatives indicated the Initiative had limited or no impact on company resourcing. In these companies, the limited impact on resourcing was most commonly due to the company’s pre-existing focus and commitment to nutrition and health or the company having an established nutrition team and associated resourcing. However, several other representatives mentioned that their companies had limited resourcing or capacity, or a very small nutrition team.

###  4. Perspectives of Company Representatives Regarding Engagement With the Public Health Community

####  4.1. Feedback on Process for BIA-Obesity Australia Initiative

 Feedback on the process for the BIA-Obesity Australia Initiative was varied, with representatives noting both strengths and suggested areas for improvement for the Initiative. In the surveys, the majority (>70%) of representatives thought that the outputs of the BIA-Obesity Australia Initiative, including identification of areas of strength, areas for improvement and good practice examples, were useful (see Figure S2). Representatives commonly reported that they liked the collaborative approach of the research, opportunity to engage with researchers, and the open communication from the researchers. Similarly, in the interviews, representatives reported that they appreciated the openness and transparency of the researchers, and an approach to benchmarking that consulted with industry.

 In the surveys as well as the interviews, representatives indicated that the process could be improved by simplifying the survey to reduce the work associated with participating, providing more detail in the scoring criteria, and providing more examples of best practice. Several representatives suggested that a longer timeframe to consult on and review the draft publication prior to public launch would be beneficial, and that this would improve the likely applicability of company-specific recommendations. Additionally, some representatives sought further engagement with the research team to better understand the findings (eg, through an additional workshop, industry working group, or other collaborative step).

####  4.2. Ways the Public Health Community Could Support Companies to Make Changes With Regards to Nutrition and Health 

 In the interviews, the most common suggestion from company representatives for the public health community to support industry change was to be more open to collaboration, communication, and engagement with industry to achieve common goals.

*“I think definitely in the area of public health, just acknowledging that the food industry is always going to be supplying food to the public and the more that we can do to work together the bigger the influence is that we will have collectively” – *Company representative (research and development role).

 Some representatives perceived unconstructive antagonism between health professionals working within industry and those working within the public health community (including government and academia). Three representatives stated that the public health community needed to be more cognisant of the limited nutrition resources within companies and the commercial constraints on making nutrition-related changes within a food and beverage company. A number of representatives desired support, advice, or technical expertise to achieve nutrition-related targets, as well as provision of compelling data and evidence to support actions.

*“When we want [nutrition-related] advice and different insights it’s very, very hard to get. It’s really hard to get because sometimes the best perspective to help you think about what you’re doing is a completely different perspective to the one [the company has]... A willingness [from the public health community] to provide input and advice… would be really helpful” – *Company representative (external/public relations/affairs role).

####  4.3. Benchmarking Effectiveness 

 In the interviews, the majority of representatives from participating and non-participating companies reported that benchmarking exercises, such as the BIA-Obesity Australia Initiative, have the potential to encourage action from the corporate sector. Representatives thought it was useful to see how they compared to other companies, and to have an external perspective on how companies were performing or being measured with regards to nutrition and health.

*“I mean benchmarking exercise is always good, because it’s something we can refer to, we know what you guys [public health community] are looking at and how we’re being measured” – *Company representative (nutrition/health/wellbeing role).

 Representatives had a number of suggestions for how benchmarking effectiveness could be maximised, including: ensuring a collaborative approach; comparing like with like (eg, taking differences in the nature of company product portfolios and local business contexts into account); and ensuring there is a long enough timeframe between assessment periods for meaningful changes to occur. Some companies suggested that the usefulness of benchmarking depended on the characteristics of the company, for example their competitive positioning with respect to health issues, and the extent of existing commitment and resourcing related to obesity prevention and population nutrition. A small number of representatives noted concerns with public benchmarking and thought that it would be more useful to focus on internal feedback to companies, including discussions with companies around their targets and strategies before they implement them.

## Discussion

 This was the first study to formally evaluate the impact of a benchmarking and accountability initiative (BIA-Obesity Australia) on food and beverage company policies and practices regarding nutrition and obesity. The BIA-Obesity Australia Initiative generated extensive visibility of nutrition issues and the role of the food and beverage industry in addressing these issues, both in the media and internally within companies. The findings from this evaluation indicate that the BIA-Obesity Australia Initiative resulted in some positive changes to internal food and beverage company policies, commitments, and disclosure practices related to obesity prevention and population nutrition, and helped to raise the priority and level of resources given to nutrition-related issues within companies, although the impact of the Initiative varied across companies.

There are a number of monitoring and accountability initiatives in the area of nutrition that target various stakeholders within the food system. Some examples include the Global Nutrition Report Country Profile,^35^ the World Bank Nutrition Country Profile,^36^ the Global Hunger Index^37^ and the Global Food Security Index.^38^ Recent research by Manorat and colleagues investigated available data visualisation tools in the area of nutrition and suggested that whilst several initiatives focused on similar nutrition topics, they were often heterogenous in the indicators and methodologies used, potentially leading to confusion and dilution of key messaging around nutrition.^39^ In order for benchmarking initiatives to be most useful to stakeholders, Manorat and colleagues identified the importance of clearly identifying the decisions and users that they are trying to influence and including actionable recommendations and appropriate dissemination formats for stakeholders.^39^ Moreover, a 2013 review of monitoring and accountability initiatives in the area of public health identified that there is limited reporting around whether monitoring and accountability initiatives have an impact on policy development and implementation.^40^

With respect to the food and beverage industry specifically, there are several benchmarking and accountability initiatives that are aimed at improving policy and practice.^15,25,41-43^ Oxfam’s ‘Behind the Brands’ initiative, which assessed food and beverage companies on social and environmental policies and practices, reported that in the three years since the initiative, companies had made significant new commitments to improve social and environmental standards in their supply chains.^44^ Similarly, Know the Chain’s ‘Food and Beverage Benchmark Findings Report,’ which benchmarks food and beverage companies in relation to forced labour in the supply chain, has also reported improvements in certain policies and practices between the 2016 and 2018 assessments.^41^ Additionally, the ATNI has reported that a number of companies involved in the assessment have improved their scores over time.^15^ However, in each of these cases, there is no evidence to indicate the extent to which the benchmarking exercises themselves contributed to observed improvements.

 To inform future benchmarking and accountability initiatives, it will be important to understand the factors that moderate the success of these initiatives as tools for driving change, including consideration of the different characteristics and contextual factors driving company behaviour.

###  Content Factors

 This evaluation of the BIA-Obesity Australia Initiative provided evidence that the provision of benchmarking data to food and beverage companies was valued by company representatives and had the potential to be useful for driving change. In particular, clear demonstration of company performance in relation to competitors, the identification of recommended areas for improvement at the company and sector level, and the inclusion of best practice examples were highlighted as valuable. Future accountability initiatives should prioritise the inclusion of these components to increase the likelihood of impact on company policies and practices. The BIA-Obesity Australia Initiative garnered extensive media coverage, which suggests strong public interest in the research area that was likely facilitated by the presentation of key findings in the format of simple numerical rankings presented in an accessible and easily understood format.

This evaluation also identified a number of areas that company representatives believed the BIA-Obesity tool and process could be improved. Companies indicated that data collection processes were relatively onerous and would benefit from being simplified. However, this may reflect more on the relatively low levels of resourcing for nutrition issues within companies, rather than on the requested volume and nature of data requested. More generally, the lack of dedicated company resourcing to respond to data collection requests of this nature reflects that companies may currently have limited sophistication in reporting on non-financial performance metrics, including on corporate sustainability issues. Whilst there has been a significant increase in corporate sustainability reporting,^45,46^ the lack of agreed national and international standards on corporate reporting regarding nutrition and health and the voluntary nature of such reporting contributes to large variability in the extent and nature of company disclosures.^47^ As more corporate sustainability initiatives, such as ATNI and BIA-Obesity, emerge there are likely to be increasing demands on companies to publicly report their performance on a range of sustainability issues. Standardisation and regulation of reporting requirements in key areas of corporate sustainability may reduce disparate demands on companies, and increase transparency and accountability.^47^

###  Contextual Factors

 This study revealed that there were a number of internal and external contextual factors that may have motivated individual companies to be more engaged in the BIA-Obesity Australia Initiative, or be more likely to make changes as a result of the Initiative.

 From an internal company perspective, those companies that had already taken substantial steps to develop and disclose policies and commitments related to obesity prevention and population nutrition, or had a focus and dedicated resourcing for nutrition, reported limited changes as a result of their involvement in the BIA-Obesity Australia Initiative. There did not appear to be a clear trend in participation and engagement between internationally versus locally headquartered companies; however, there was low level of engagement from companies in the quick service restaurant sector. As part of their process of engagement with food and beverage company representatives, the research team drew on their existing relationships with public health groups and researchers that already engaged with the food and beverage industry. These existing relationships may have facilitated company engagement in the BIA-Obesity Australia Initiative. Interestingly, the nature and healthiness of the company’s product portfolio did not appear to influence the degree to which they engaged with or responded to the Initiative. Future initiatives in this area could explore the value of providing additional support (eg, nutrition expertise, category level nutrition benchmarking, change management advice) to companies that have made limited progress to address nutrition concerns.

From an external perspective, pressure from society and government have been shown to be key contextual contributors to change within an organisation.^27,48^ The BIA-Obesity Australia Initiative attracted extensive media coverage at a local and national level, which was noted by representatives in the evaluation. This high level of media coverage is likely to have contributed to the level of exposure the Initiative had, particularly at a senior management level within companies. The nature of benchmarking, in which companies are compared to one another in a simple format that is easily communicated, may have facilitated media uptake, and therefore helped garner public and industry attention to nutrition issues. Conversely, at the time of the Initiative, there appeared to be limited pressure from government on the food and beverage industry to take positive action. For example, the Australian Government’s key nutrition policies at the time (the Health Star Rating scheme,^49^ a front-of-pack interpretive labelling system, and the Healthy Food Partnership,^50^ a private-public partnership) were voluntary in nature, with limited credible threat of legislative change in the area of nutrition. This may have limited company motivation to enact changes at the time.

This evaluation of the BIA-Obesity Australia Initiative should be considered in the context of the well-documented strategies used by the food and beverage industry to influence policy development and public opinion in their favour.^10,12,51,52^ These strategies include ‘policy substitution’ in which voluntary commitments from companies are adopted as part of efforts to delay or circumvent mandatory regulations from government.^10^ It could be argued that the BIA-Obesity Australia Initiative’s focus on voluntary commitments may distract attention from the need for greater government action to address unhealthy diets.^2,5,53^ This is of particular concern given evidence of the weakness of current industry self-regulatory approaches in improving the healthiness of food environments.^54-56^ However, as noted in key reports recommending strategies to improve population diets,^2,14^ a whole-of-society approach is needed, and a focus on progress in one area need not preclude increased action in other areas. Whilst voluntary approaches alone have proved insufficient to drive meaningful action for nutrition,^51^ benchmarking can still play a role in increasing private sector accountability and improving the quality of corporate responsibility reporting.^51,57^ In addition, the BIA-Obesity Australia Initiative highlights the value of benchmarking to expose and quantify the differences in the strength of policies and commitments amongst the included food and beverage companies.

###  Process Factors 

There were several themes identified related to the process of generating and maintaining change within companies. Regarding media exposure, the research team spent a substantial amount of time liaising with a professional media team which likely facilitated the media coverage generated by the Initiative. Representatives that participated in the in-depth interviews most commonly reported having nutrition, health or wellbeing roles within their company, and several representatives were in senior positions within the business. A number of representatives also reported that senior business had been supportive or engaged in the Initiative, often due to proactive efforts of the representatives involved in the study. Advocates for change within an organisation, as well as strong leadership, have been shown to be fundamental to the success of change initiatives,^27,48,58^ and engaging these advocates as part of a monitoring and accountability initiative is likely to increase its impact. Future initiatives should explicitly consider ways to further engage senior representatives within companies to increase buy-in and potential for organisational change.

This evaluation indicated the perceived importance of repeating these types of initiatives to monitor progress over time and maintain pressure on companies to take positive action. This is consistent with the findings from other benchmarking and accountability initiatives, such as Know the Chain and the ATNI, which have shown that an ongoing monitoring approach can lead to increased engagement and improvements to policy and practice over time.^41,15^ Monitoring over time is also important for acknowledging progress made by companies, and in reflecting efforts made in response to benchmarking and accountability initiatives.

Benchmarking and accountability initiatives, such as BIA-Obesity Australia, that involve engagement with the food and beverage industry are vulnerable to the ‘constituency building’ strategy of the food and beverage industry, whereby companies endeavour to establish relationships with key stakeholders (including public health researchers) in order to gain favour, influence and build credibility.^10^ In light of the perceived value of constituency building to food and beverage companies, it is perhaps unsurprising that company representatives highlighted the high level of engagement between the researchers and company representatives as a strength of the Initiative, and indicated their support for increased collaboration, communication and engagement between the public health community and industry in future initiatives. Nevertheless, representatives articulated that engagement with the research team enabled the recommendations in the report to be more tailored to their context, which may have increased the likelihood that these recommendations could be implemented. Moreover, the research team invested substantial time in company engagement and communication throughout the BIA-Obesity Australia Initiative. This included hosting a workshop for company representatives to present preliminary findings, providing advanced copies of the media release and final reports to companies, and conducting in person meetings with company representatives where requested. This process is likely to have facilitated company engagement in the Initiative, as well as more accurate documentation of company policies and understanding of ways in which the Initiative could be improved. Provided strong processes are in place to minimise and manage potential conflicts of interest and ensure the independence of the assessment process, there are likely to be important benefits from continuing engagement.

Several companies responded negatively to the process. This included a threat of litigation to members of the research team – a strategy that has not previously been identified as part of analyses of food and beverage industry corporate political activity in Australia.^12,52^ Researchers undertaking similar initiatives in future would benefit from anticipating such responses, and taking steps (such as setting up project governance structures, and obtaining legal advice prior to public release of findings) to reduce the potential impact of such threats.

More generally, it will be important for future initiatives to mitigate and minimise potential risks associated with engagement with the food and beverage industry throughout the process.^59^ Mechanisms for this could include: ensuring independent funding for the project (as was the case for the BIA-Obesity Australia Initiative), engaging with an independent organisation to liaise with companies, and having an independent steering committee in place to oversee the research process.^21^ Future initiatives should also formally include government engagement and dialogue with policy-makers as part of the benchmarking process, particularly in light of strong evidence demonstrating the ineffectiveness of a voluntary, industry-guided approach to tackling population nutrition and obesity.^54-56,60-62^ Where performance of industry is shown to be limited (such as in the case reported here), these initiatives could serve to highlight the need for greater government regulation in nutrition and obesity prevention. Additionally, governments can play a role in strengthening industry self-regulatory commitments, through implementing strong regulatory reporting frameworks; time-bound, measurable targets for food and beverage industry action; and sanctions for non-compliance.^63^ In Australia, the Healthy Food Partnership (HFP),^50^ a collaboration between the government, the public health sector and the food and beverage industry, could represent a potential vehicle for implementing such policies. The HFP work program already includes development of product reformulation targets, and could be expanded to include additional focus areas and greater incentives for compliance with targets.^50^ Importantly, the HFP has been criticised for making slow and limited progress^56^ and it would require stronger political leadership from Australian politicians to implement meaningful regulatory change in the area of population nutrition.

###  Strengths and Limitations

 This evaluation had several strengths. As far as the authors are aware, this was the first formal evaluation of a food and beverage industry benchmarking and accountability initiative related to obesity prevention and population nutrition. It provided evidence of the value of these initiatives for provoking incremental improvements in food and beverage industry nutrition policies and practices. The evaluation used a mixed-methods design to triangulate data from several sources and was conducted over a relatively long timeframe (16 months) to evaluate short- and medium-term outcomes of the Initiative. The evaluation was somewhat limited by the response rate; 11 out of 28 invited companies completed the evaluation survey, and 16 out of 28 invited companies participated in the interviews, with low participation from the quick service restaurant sector and non-participating companies. The evaluation was also influenced by the number of companies that originally participated in the BIA-Obesity Australia Initiative (n = 16/34). The participation rates may have introduced some participation bias and may narrow the generalisability of the evaluation results to primarily those companies that were willing to participate in the research. Conversely, participation in the evaluation may have been positively impacted by the strong media coverage and exposure associated with the BIA-Obesity Australia Initiative. In relation to the media coverage, whilst a high-level content analysis of media articles was conducted, a detailed categorisation of industry and other stakeholder media responses in relation to the Initiative was not conducted. Finally, this evaluation focused on food and beverage industry perspectives, as the primary participant group in the Initiative. It did not consider other stakeholder perspectives, such as those of government, the public health community or consumers. It will be important to include these stakeholders as part of future evaluation studies to capture a broader range of opinions on the effectiveness of these types of initiatives.

## Conclusion

 In light of slow progress to address unhealthy diets and obesity, benchmarking and accountability initiatives are one mechanism to motivate change and encourage meaningful action from the food and beverage industry towards improving the healthiness of food environments. The findings from this evaluation indicate that engagement with food companies through a benchmarking and accountability initiative can lead to changes in nutrition-related policies, resourcing and disclosure practices, as reported by company representatives. Importantly, the benefits of industry accountability initiatives need to be assessed in the context of the broader political and economic environment. Particular attention must be paid to circumstances where industry is known to use voluntary self-regulation to delay or circumvent mandatory regulations. Provided that benchmarking standards are based on evidence-based public health recommendations, as is the case with BIA-Obesity, accountability initiatives provide an opportunity for the performance of the industry to be assessed in an objective way. Where industry performance is found to be weak, as was the case in the BIA-Obesity Australia Initiative, accountability initiatives can play an important role in drawing attention to the need for increased government regulation. Further evaluations of accountability initiatives in the area of nutrition and obesity are needed to understand the longer-term effects of these approaches from different stakeholder perspectives and in different regulatory contexts.

## Acknowledgments

 GS and MB are researchers within a National Health and Medical Research Council (NHMRC) funded Centre of Research Excellence in Food Retail Environments for Health (RE-FRESH) (APP1152968). GS is also a researcher within a NHMRC Centre for Research Excellence entitled Reducing Salt Intake Using Food Policy Interventions (APP1117300).

## Ethical issues

 The research was approved by the Human Ethics Advisory Group (HEAG) of the Faculty of Health at Deakin University, Australia.

## Competing interests

 Authors declare that they have no competing interests.

## Authors’ contributions

 ER contributed to the conceptualisation of the study, study design, data collection, data analysis and drafting the manuscript. MB contributed to the study design and data analysis. GS contributed to the conceptualisation of the study, study design, data analysis and drafting the manuscript. All authors critically reviewed and edited the manuscript.

## Funding

 GS is supported by a Heart Foundation Future Leader Fellowship (102035) from the National Heart Foundation of Australia. MB is supported by a post-doctoral fellowship from the Institute for Health Transformation, Deakin University. The funders had no role in study design, data collection and analysis, decision to publish, or preparation of the manuscript.

## Supplementary files


Supplementary file 1 contains Appendix S1, Figures S1-S2, and Tables S1-S4.
