# Supplementary material for: Benchmarking Food and Beverage Companies on Obesity Prevention and Nutrition Policies: Evaluation of the BIA-Obesity Australia Initiative, 2017-2019
Source: Int J Health Policy Manag. 2020 Aug 22;10(12):857–70. doi: 10.34172/ijhpm.2020.147 (PMC9309961; doi:10.34172/ijhpm.2020.147)
Supplement: Supplementary file 1 — contains Appendix S1, Figures S1-S2, and Tables S1-S4. [file ijhpm-10-857-s001.pdf]

Supplementary file 1

Appendix S1. Company response to the BIA-Obesity Australia Initiative in the media

Following the launch of the 'Inside our Supermarkets' report, Woolworths publicly reaffirmed their commitment to customer health and responded that "While the Report highlights some of the progress we have made, we do acknowledge there is more we can do." [1] Coles noted that they were committed to nutrition and health, and that "the report does not take account of the important role of exercise in a healthy lifestyle."[1] IGA and ALDI acknowledged their role as retailers in promoting nutrition and health, however raised concerns around the report not adequately taking into account actions that they were taking in this area.[1] Several companies also responded to the release of the 'Inside our Food and Beverage Manufacturers' report, with some non-participating companies such as Schweppes and Kraft Heinz noting actions they had already taken to improve population nutrition (eg, in the area of reformulation). Several participating companies, including Fonterra and Coca-Cola, released media statements that noted the findings of the report and outlined their role in addressing population nutrition and obesity in Australia.[2,3] Other companies, such as McCain, expressed concerns around the project methods when asked for public comments by the media in response to the report.[4] In response to the 'Inside our Quick Service Restaurants' report, Domino's Australia released a YouTube video in which their group Chief Executive Officer outlined their commitment to 'healthier and tastier menu options' and noted that they already meet a number of the recommendations highlighted in the report.[5,6] Grill'd publicly denounced the quality of the report and commented in the media that they were considering legal action against the report authors.[5]

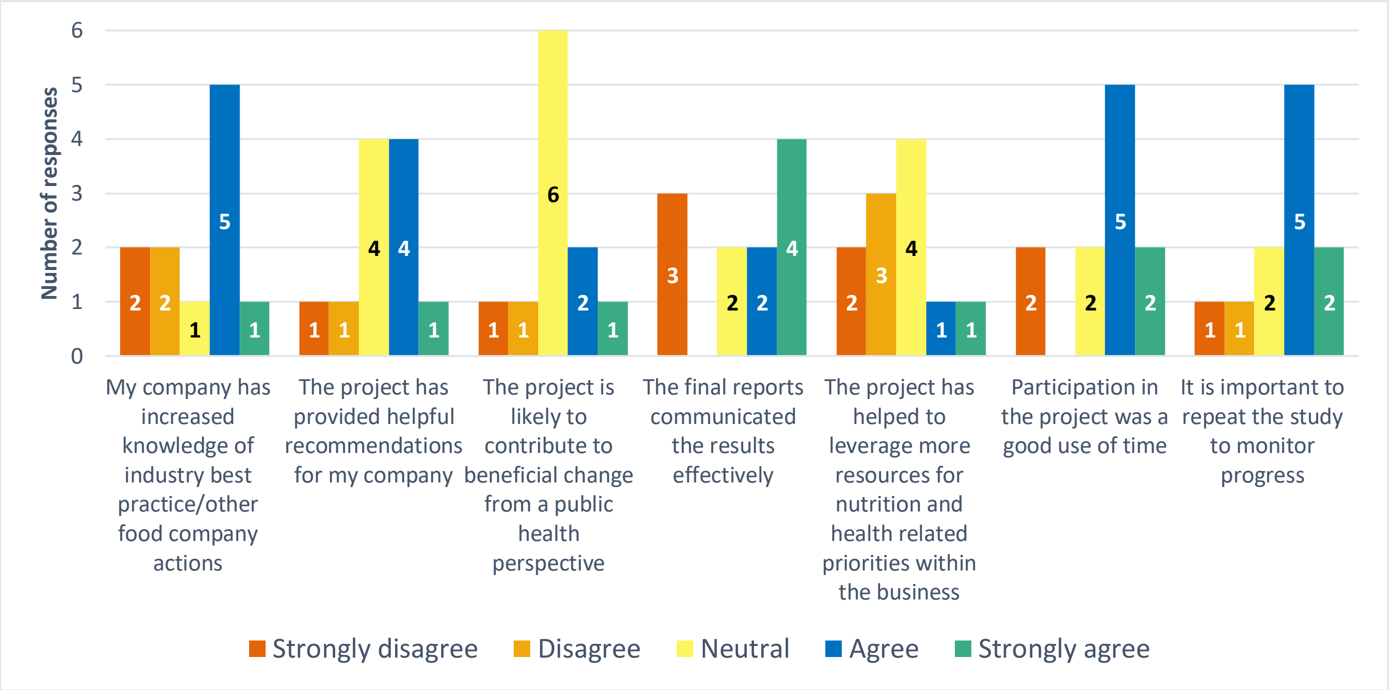

Figure S1. Views of company representatives on outcomes from the BIA-Obesity Australia Initiative (from survey) (n=11 companies). Abbreviation: BIA-Obesity, Business Impact Assessment – Obesity and population nutrition.

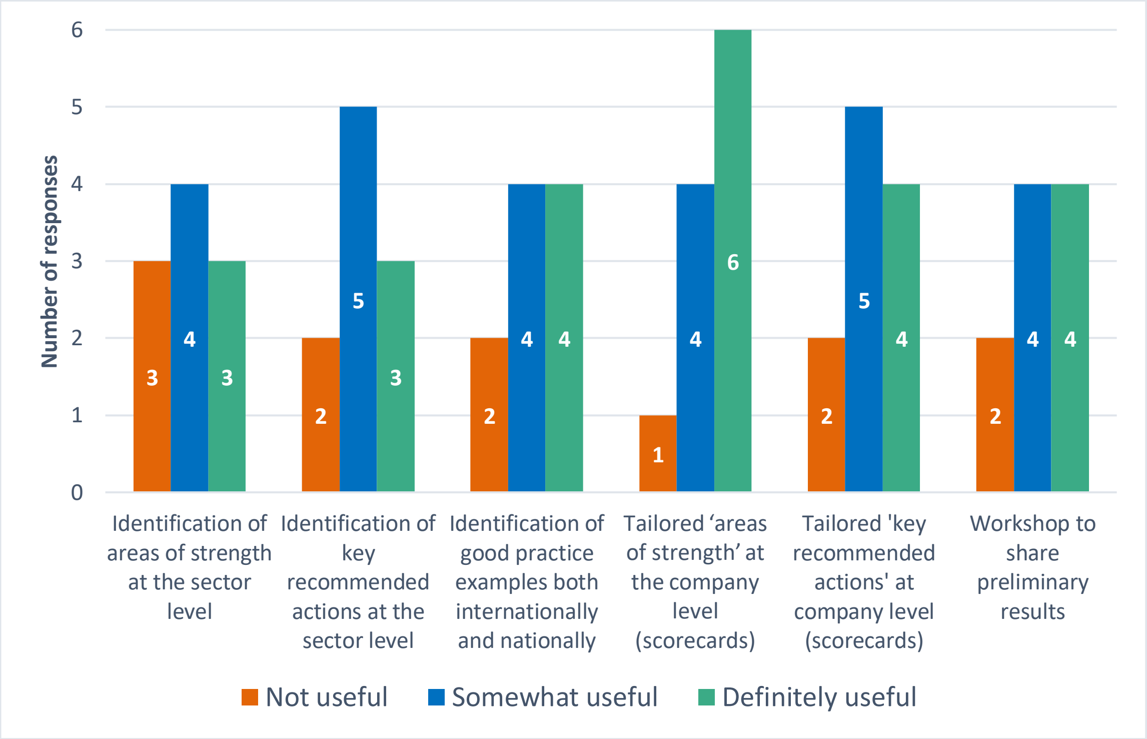

Figure S2. Views of company representatives on the usefulness of outputs from the BIA-Obesity Australia Initiative (from survey) (n=11 companies). Abbreviation: BIA-Obesity, Business Impact Assessment – Obesity and population nutrition.

Table S1. Logic model for the BIA-Obesity Initiative[1]

Problem	Inputs	Outputs (activities)	Outputs (participation)	Outcomes (short-term: 1-3 months)	Outcomes (medium-term: 6-12 months)	Outcomes (long-term: 3-5 years)
**Obesity and unhealthy diets are a leading cause of global mortality and morbidity, driven by increasingly unhealthy food environments. The United Nations and the World Health Organization identify the food and beverage industry as playing a key role in contributing to the creation of healthier food environments. Monitoring frameworks are needed to increase transparency and accountability from the food and beverage industry in**	• Timeframe for completion: 12-18 months<br>• Project team resources: one junior researcher (full-time), one senior researcher (one day per week)<br>• Company representative resources (staff time to complete survey and liaise with project team)<br>• INFORMAS international members: ad hoc support for country-level project team, and dual assessment tasks<br>• Steering committee: occasional input and oversight	• Select companies for inclusion<br>• Tailor tool to country context<br>• Collect relevant publicly-available company information<br>• Liaise with companies to explain process and obtain additional information<br>• Assess companies using the BIA-Obesity tool<br>• Prepare scorecards for each company, including recommendations<br>• Share results with companies privately<br>• Publicly release results, including associated media and resources<br>• Evaluate impact - survey evaluation, active	• Target companies: food and beverage companies with leading shares in each of the food and non-alcoholic beverage manufacturing, food retail, and restaurant sectors, as well as relevant industry associations<br>• Contacts within companies: leads of nutrition, corporate responsibility and / or external affairs<br>• Public health-related organisations and professional associations can support the project to align messaging and increase impact	• Engage food and beverage companies in issues related to nutrition and health<br>• Identify best practice examples, both at the country level and internationally<br>• Identify areas for improvement across each sector of the food and beverage industry, as well as at the company level<br>• Increase media and public attention on the role of the food and beverage industry in contributing to the creation of healthier food environments	• Drive increased accountability from food and beverage companies<br>• Increase company transparency related to nutrition and health activities<br>• Leverage more resources for nutrition and health teams within companies, to address areas for improvement identified by the project<br>• Increase engagement from food and beverage companies in the research process for future iterations	• Food and beverage companies meet best practice standards across key policy domains related to obesity prevention and population nutrition<br>• Establish sustained monitoring of the food and beverage industry in countries, comparing changes across time<br>• Improve the healthiness of the food environment and reduce the burden of overweight and obesity

**addressing obesity and related nutrition issues.**	• Graphic designer to design final reports and printing (optional)	monitoring of company websites<br>and media evaluation<br>• Conference presentations and academic publications	• Government representatives can support the project to align/support existing government policies (where relevant)		• Use the project outcomes to secure resources for follow up assessment	• Embed nutrition-related metrics as part of corporate sustainability frameworks and investment decisions

Abbreviations: BIA-Obesity, Business Impact Assessment – Obesity and population nutrition; INFORMAS, International Network for Food and Obesity/NCDs Research.

[1] Previously published in Sacks G, Vanderlee L, Robinson E, et al. BIA-Obesity (Business Impact Assessment—Obesity and population-level nutrition): A tool and process to assess food company policies and commitments related to obesity prevention and population nutrition at the national level. *Obesity Reviews.* 2019;0(0). doi:10.1111/obr.12878. Reproduced with permission from the authors.

Table S2. BIA-Obesity Australia Initiative Evaluation Survey

1. What sector of the food industry do you primarily work for? (choose one)
   Food/beverage manufacturer **Supermarket** **Quick Service Restaurant**
2. In your opinion, how useful were each of the following outputs of the project (please rate each)
   a. Identification of areas of strength at the sector level
      Definitely useful **somewhat useful** **not useful**
   b. Identification of key recommended actions at the sector level
      Definitely useful **somewhat useful** **not useful**
   c. Identification of good practice examples both internationally and nationally
      Definitely useful **somewhat useful** **not useful**
   d. Tailored 'areas of strength' at the company level (scorecards)
      Definitely useful **somewhat useful** **not useful**
   e. Tailored 'key recommended actions' at the company level (scorecards)
      Definitely useful **somewhat useful** **not useful**
3. Please rate your level of agreement with the following statements relating to the BIA-Obesity Australia project *(please mark one box for each row)*:

**As a result of participation in the project (outcome):**	**Strongly disagree**	**Disagree**	**Neutral**	**Agree**	**Strongly agree**
a) My company has increased knowledge of industry best practice/what other food companies are doing both nationally and internationally as a result of this project					
b) The project has provided helpful recommendations for my company					
c) Participation in the project was a good use of my time / that of my colleagues					
d) The final reports communicated the results of the project effectively					
e) The project has helped to leverage more resources for nutrition and health related priorities within the business					
f) The project is likely to contribute to beneficial change from a public health perspective					

g) It is important to repeat the study in order to monitor progress					

4. In your view, when would be an appropriate time to repeat the process?
   1 year 2 years 3 years >3years Not at all
5. If we repeated the BIA-Obesity project in Australia, would your company be willing to participate again?
   (Yes/no)
6. Please provide comments on what you liked about the BIA-Obesity Australia project.
7. Please comment on how the results of the project were received within your company (eg, generally seen as a worthwhile exercise, not really noticed at all, seen in a negative light).
8. In what ways (if any) has the BIA-Obesity Australia project influenced your work or the policies / actions of your company (for example, changes to priorities, plans, practices, resources, etc)?
9. Please provide suggestions for how we could improve the process in future iterations (if any).
10. Please provide any additional comments about your experience in relation to the BIA-Obesity Australia project.

Abbreviation: BIA-Obesity, Business Impact Assessment – Obesity and population nutrition.

Table S3. BIA-Obesity Australia Initiative Interview Guide

**Introduction and purpose**<br>*Thank you for taking the time to speak with me today. As a participant in the 2017/18 BIA-Obesity Australia initiative, you will be aware that our team at Deakin University conducted a project last year that assessed major Australian food companies on their policies related to nutrition and obesity prevention. We are conducting interviews with participating company representatives as part of a 12 month outcome evaluation. This data will be used to understand the impacts of the project on your company, and how future iterations of the project could be improved. Your responses to these interview questions will be reported anonymously in any publications or other public reporting that results from this work. With your consent, the interview will be audio recorded.*
**Question**
1. What is your role in the company, and how long have you been in this role?
2. What involvement did you have in the BIA-Obesity Australia project?
3. To what extent was the project noticed within your company?<br>a. At what levels was it noticed and by whom? How was it used?<br>b. Was it noticed and used just around the launch of the results, or has it been used subsequently?<br>c. What parts of the report were most noticed? For example, the individual scorecard for your company, the recommendations, the comparison to others?
4. Do you think that being involved in the BIA-Obesity Australia project has led to any changes in your company regarding its approach to addressing nutrition and obesity prevention issues? If so:<br>a. How do you think your company's approach to nutrition and obesity prevention has changed since the project?<br>b. What aspect of the project contributed to that?<br>c. What else has changed in response?
5. One of the areas of focus for the project was on increasing transparency. Do you think that being involved in this project led to any changes in the level of disclosure / transparency of your company in relation to its policies and commitments related to nutrition and obesity prevention?
6. Thinking about independent benchmarking exercises (eg, ones in health, the environment, sustainability) in general. How effective do you think they are at encouraging more corporate action in a particular area?<br>a. What do you think makes some more effective than others?
7. Were there things that you believe could have been done better in BIA-Obesity Australia to try to encourage more action from your company?
8. What else do you think could be done by the public health community to support your company to make further changes?
9. Is there anything else that you would like to add in relation to your company's experience with the BIA-Obesity Australia project?

Abbreviations: BIA-Obesity, Business Impact Assessment – Obesity and population nutrition

Table S4. Views of company representatives on when to repeat the BIA-Obesity Australia Initiative and willingness to participate in future assessments (from survey) (n=11 companies)

**In your view, when would be an appropriate time to repeat the process?**	**Number of responses (%)**
1 year	2 (18%)
2 years	3 (27%)
3 years	2 (18%)
>3 years	3 (27%)
Not at all	1 (9%)
Total responses	11
**If we repeated the BIA-Obesity Initiative in Australia, would your company be willing to participate?**	
Yes	9 (90%)
No	1 (10%)
Total responses	10

References

1. SBS News. Supermarkets respond to calls to act on nation's obesity problem. 2018; https://www.sbs.com.au/news/supermarkets-respond-to-calls-to-act-on-nation-s-obesity-problem. Accessed October, 2019.

2. Coca Cola Company. Our role in tackling obesity (updated August 2019). 2018; https://www.coca-colacompany.com/au/news/coca-cola-sugar-reduction.html. Accessed November, 2018.

3. Fonterra. Our commitment to nutrition. 2018; https://www.fonterra.com/au/en/our-stories/media/media-statement-our-commitment-to-nutition.html. Accessed November, 2018.

4. The Sydney Morning Herald. Laziest food companies exposed in scathing 'obesity prevention' report card. 2018; https://www.smh.com.au/national/laziest-food-companies-exposed-in-scathing-obesity-prevention-report-card-20180322-p4z5oq.html. Accessed October, 2019.

5. The Sydney Morning Herald. 3/100: The fast-food company with the worst health score. 2018; https://www.smh.com.au/national/nsw/3-100-the-fast-food-company-with-the-worst-health-score-20180527-p4zhrw.html. Accessed August, 2018.

6. Dominos Australia. Group CEO Don Meij discusses healthier, tastier menu options. 2018; www.youtube.com/watch?v=NOZQwThwlSs&feature=youtu.be. Accessed August, 2018.
